# Global Trends in CD4 Count Measurement and Distribution at First Antiretroviral Treatment Initiation

**DOI:** 10.1093/cid/ciae548

**Published:** 2024-11-06

**Authors:** Reneé de Waal, Kara Wools-Kaloustian, Ellen Brazier, Keri N Althoff, Antoine Jaquet, Stephany N Duda, Nagalingeswaran Kumarasamy, Theodora Savory, Helen Byakwaga, Gad Murenzi, Amy Justice, Didier K Ekouevi, Carina Cesar, Mark K U Pasayan, Agness Thawani, Charles Kasozi, Pelagie Babakazo, Maile Karris, Eugene Messou, Claudia P Cortes, Cordelia Kunzekwenyika, Jun Yong Choi, Noela C Owarwo, Annabelle Niyongabo, Vincent C Marconi, Oliver Ezechi, Jessica L Castilho, Kathy Petoumenos, Leigh F Johnson, Nathan Ford, Reshma Kassanjee

**Affiliations:** Centre for Infectious Disease Epidemiology and Research, School of Public Health, University of Cape Town, Observatory, South Africa; Department of Medicine, Indiana University School of Medicine, Indianapolis; Institute for Implementation Science in Population Health, City University of New York, New York; Department of Epidemiology, Bloomberg School of Public Health, Johns Hopkins University, Baltimore, Maryland; National Institute for Health and Medical Research UMR 1219, Research Institute for Sustainable Development EMR 271, Bordeaux Population Health Research Centre, University of Bordeaux, France; Department of Biomedical Informatics, Vanderbilt University Medical Center, Nashville, Tennessee; Infectious Diseases Medical Centre, Chennai Antiviral Research and Treatment Clinical Research Site, Voluntary Health Services, Chennai, India; Centre for Infectious Disease Research in Zambia, Lusaka; Department of Community Health, Mbarara University of Science and Technology, Uganda; Einstein-Rwanda Research and Capacity Building Program, Research for Development, and Rwanda Military Referral and Teaching Hospital, Kigali; Veterans Affairs Connecticut Healthcare System, Yale Schools of Medicine and Public Health, Yale University, New Haven; Centre de Formation et de Recherche en Santé Publique, Université de Lomé, Togo; Research Department, Fundacion Huesped, Buenos Aires, Argentina; Research Institute for Tropical Medicine, Muntinlupa City, Philippines; Lighthouse Trust, Lilongwe, Malawi; Masaka Regional Referral Hospital, Masaka City, Uganda; Kinshasa School of Public Health, University of Kinshasa, Democratic Republic of the Congo; Department of Medicine, University of California, San Diego; Centre de Prise en charge, de Recherche et de Formation Yopougon-Attié, Abidjan, Côte d’Ivoire; Department of Internal Medicine, Faculty of Medicine, University of Chile, and Hospital Clínico San Borja Arriarán and Fundación Arriarán, Santiago; SolidarMed Zimbabwe, Masvingo; Division of Infectious Diseases, Department of Internal Medicine, Yonsei University College of Medicine, Seoul, South Korea; Infectious Diseases Institute, Makerere University, Kampala, Uganda; Association Nationale de Soutien aux Séropositifs et malades du SIDA-Santé PLUS, Bujumbura, Burundi; Division of Infectious Diseases, School of Medicine and Hubert Department of Global Health, Rollins School of Public Health, Emory University, Atlanta, Georgia; Centre for Reproduction and Population Health Studies, Nigerian Institute for Medical Research, Lagos; Division of Infectious Diseases, Vanderbilt University Medical Center, Nashville, Tennessee; The Kirby Institute, University of New South Wales, Sydney, Australia; Centre for Infectious Disease Epidemiology and Research, School of Public Health, University of Cape Town, Observatory, South Africa; Centre for Infectious Disease Epidemiology and Research, School of Public Health, University of Cape Town, Observatory, South Africa; Department of Global HIV, Hepatitis and STI Programmes, World Health Organization, Geneva, Switzerland; Centre for Infectious Disease Epidemiology and Research, School of Public Health, University of Cape Town, Observatory, South Africa

**Keywords:** CD4 at ART start, Advanced HIV disease, HIV care, Global testing trends, Treat-All

## Abstract

**Background:**

While people with human immunodeficiency virus (PWH) start antiretroviral treatment (ART) regardless of CD4 count, CD4 measurement remains crucial for detecting advanced human immunodeficiency virus (HIV) disease and evaluating ART programs. We explored CD4 measurement (proportion of PWH with a CD4 result available) and prevalence of CD4 <200 cells/µL (hereafter “CD4 <200”) at ART initiation within the International epidemiology Databases to Evaluate AIDS (IeDEA) global collaboration.

**Methods:**

We included PWH at participating ART programs who first initiated ART at age 15–80 years during 2005–2019. We described proportions of PWH with a CD4 result (measured within 6 months before to 2 weeks after ART initiation) and, among those with a CD4 result, with CD4 <200, by year of ART initiation and region.

**Results:**

We included 1 355 104 PWH from 42 countries in 7 regions; 63% were female. The median (interquartile range) age at ART initiation was 37 (3144) years in males and 32 (26–39) years in females. CD4 measurement initially increased, or remained stable over time until around 2013, but then declined to low levels in some regions (Southern Africa, except South Africa: from 54% to 13%; East Africa: 85% to 31%; Central Africa: 72% to 20%; West Africa: 91% to 53%; and Latin America: 87% to 56%). Prevalence of CD4 <200 declined over time in all regions, but plateaued after 2015 at ≥30%.

**Conclusions:**

CD4 measurement has declined sharply in recent years, especially in sub-Saharan Africa. Among those with a CD4 measurement, the prevalence of CD4 <200 remains concerningly high. Scaling up CD4 testing and securing adequate funding are urgent priorities.

## Teaser

Despite its critical role in patient care and program evaluation, measurement of CD4 cell count at treatment start has declined to low levels, especially in sub-Saharan Africa. Prevalence of CD4 <200 cells/μL among those tested has plateaued at high levels in recent years.

## Graphical Abstract

**Graphical Abstract:**
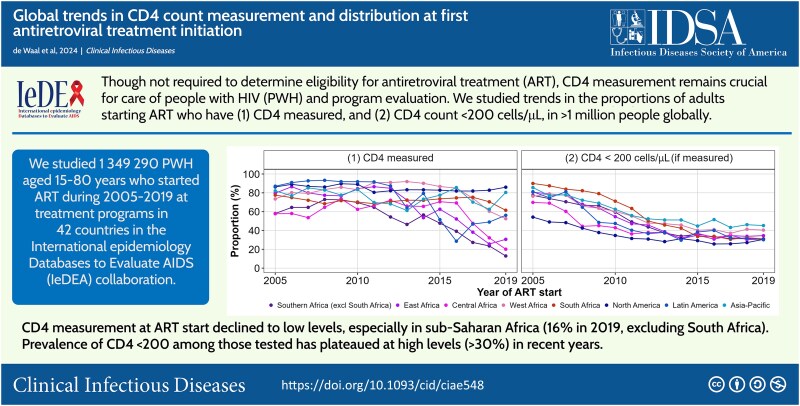
This graphical abstract is also available at Tidbit: https://tidbitapp.io/tidbits/global-trends-in-cd4-count-measurement-and-distribution-at-first-antiretroviral-treatment-initiation?utm_campaign=tidbitlinkshare&utm_source=ITP

Since 2015, the World Health Organization (WHO) has recommended immediate provision of antiretroviral treatment (ART) for all people with human immunodeficiency virus (HIV) regardless of CD4 cell count, a guideline known as “Treat-All” [1]. Although low CD4 is no longer a prerequisite for starting ART, the WHO still recommends measuring CD4 counts at first ART initiation, or reinitiation after disengagement from care, to guide individual care and to identify gaps in testing and linkage to care at the country and program level [1, 2]. Late ART initiation (CD4 count of <200 cells/µL at ART initiation) is a national priority indicator in the WHO strategic information guidelines, and its monitoring requires CD4 measurement at ART initiation [2]. Similarly, assessment of advanced HIV disease (AHD, defined as a CD4 count <200 cells/µL [hereafter “CD4 <200”] or presence of a WHO stage 3 or 4–defining condition) relies in part on CD4 measurement [1]. AHD is associated with increased risk of morbidity and mortality [3, 4], and identification of people with HIV (PWH) with AHD provides an opportunity to offer a more intensive package of care, including opportunistic infection screening and prophylaxis [1].

Despite its importance for individual care and programmatic monitoring, measurement of CD4 count has decreased sharply since the implementation of Treat-All recommendations, particularly in low- and middle-income countries [5, 6]. This has been partly attributed to reallocation of funding to measurement of HIV type 1 (HIV-1) RNA [7, 8]. Although the burden of AHD has generally declined over time, studies from Botswana, Uganda, South Africa, and China have reported persistently high prevalences, even in the Treat-All era [6, 9–11].

We describe regional trends over time in measurement of CD4 count at first ART initiation among 1.35 million adult PWH in 42 countries at treatment programs within the global International epidemiology Databases to Evaluate AIDS (IeDEA) collaboration [12–15]. Among those who had CD4 count results available, we further describe the distribution of CD4 counts at ART initiation over time.

## METHODS

We included all PWH aged 15–80 years at documented first ART initiation during 2005–2019 at treatment sites participating in the IeDEA global collaboration (www.iedea.org). We excluded those initiating ART during the coronavirus disease 2019 (COVID-19) pandemic (data from 2020 and later) because of potential data reporting delays or gaps during the COVID-19 pandemic. Our dataset did not include data from after the pandemic. We also excluded PWH with HIV RNA <1000 copies/mL at first ART initiation (as this likely represents evidence of prior HIV treatment and thus an inaccurate first ART initiation date), resulting in 2.7% of PWH being excluded.

We described the proportions of PWH who had a documented CD4 count result at the time of first ART initiation (defined as the CD4 count result closest to, and measured within 6 months before until 2 weeks after, the date of first ART initiation), referred to as “CD4 measurement” hereafter. To account for possible date errors or delays in recording CD4 results, we performed sensitivity analyses extending this definition to include CD4 results within 4 or 6 (rather than 2) weeks after ART initiation. Among those with CD4 count measured, we summarized CD4 count results, in cells per microliter, as proportions <200, and medians and percentiles, referred to as “CD4 distribution” below. We reported the above CD4 measurement and distribution statistics by year of ART initiation and region. We stratified estimates by age and birth sex in supplementary analyses.

While our study aimed to report the CD4 distribution specifically in PWH who had CD4 measured at ART initiation, it is also of public health interest to determine whether the prevalence of CD4 <200 is concerningly high in all PWH initiating ART. We performed an additional simplistic exploratory supplementary analysis using WHO staging data to produce a lower-bound estimate (ie, “best-case” scenario estimate) for the prevalence of CD4 <200 in all PWH starting ART for 2016–2019, the period after Treat-All implementation, including regions with reasonable completeness of WHO stage data at ART initiation (all regions included in the analysis had WHO stage data available for 63%–84% of eligible PWH; regions excluded had data available for 0–38% of eligible PWH). In PWH without a documented CD4 count, we assumed the same prevalence of CD4 <200 as observed in PWH with a CD4 count after matching on region, birth sex, age, year of ART initiation, and WHO stage when known; we assigned a CD4 ≥200 cells/μL when WHO stage was unknown.

Within IeDEA, each participating site has institutional review board approval to collect and share de-identified routine clinical data. Each region has approval to consolidate and analyze data.

## RESULTS

**Figure 1.**
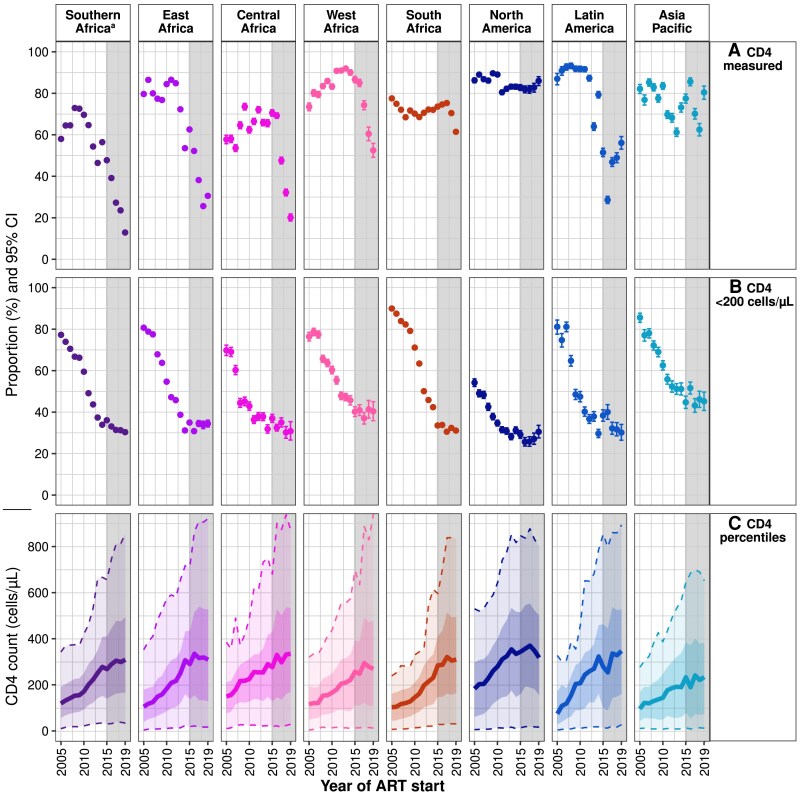
CD4 measurement and distribution at antiretroviral treatment (ART) initiation in people with human immunodeficiency virus (PWH) at International epidemiology Databases to Evaluate AIDS (IeDEA)–participating treatment programs: percentage of PWH initiating ART for whom there is a documented CD4 measurement (*A*); among those with a documented CD4 measurement, the percentage with CD4 count <200 cells/μL (*B*); and, among those with a documented CD4 measurement, the CD4 count median (solid line), first to third quartiles (darker shaded ribbon), and 5th and 95th percentiles (dashed lines) (*C*). Shaded gray areas represent the “Treat-All” era from September 2015 based on World Health Organization guidelines. ^a^Excluding South Africa. Abbreviations: ART, antiretroviral treatment; CI, confidence interval.

We included 1 349 290 PWH initiating ART from 8 “regions” (7 regions and 1 country): South Africa (316 394), the rest of Southern Africa excluding the country of South Africa (602 948), East Africa (236 981), Central Africa (51 667), West Africa (37 956), North America (51 469), Latin America (27 429), and the Asia-Pacific region (24 446). Females comprised 64%–69% of those initiating ART in the Africa regions, 17% in North America, 45% in Latin America, and 35% in Asia-Pacific (Table 1). Overall, the median age at ART initiation was 37 years (interquartile range [IQR], 31–44 years) in males and 32 years (IQR, 26–39 years) in females. Characteristics of the PWH by year of ART initiation are reported in Section S1 of the Supplementary Material, as well as the numbers underlying Figure 1, discussed below.

**Table 1.** Characteristics of 1 355 104 People With Human Immunodeficiency Virus Aged 15–80 Years Starting Antiretroviral Treatment During 2005–2019 in International epidemiology Databases to Evaluate AIDS (IeDEA)–Participating Treatment Programs, by Region/Country

Characteristic	Southern Africa^a^	East Africa	Central Africa	West Africa	South Africa	North America	Latin America	Asia-Pacific
No. of PWH starting ART	602 948	236 981	51 667	37 956	316 394	51 469	27 429	24 446
No. of countries contributing data	5	3	5	5	1	2	6	15
No. of treatment programs (within countries)	8	8	22	7	8	23	6	26
Female sex, No. (%)	386 849 (64)	154 744 (65)	33 862 (66)	25 241 (67)	218 294 (69)	8574 (17)	12 279 (45)	8500 (35)
Age at ART initiation, y, median (IQR)								
Females	31 (26–38)	33 (27–40)	34 (28–41)	34 (29–41)	31 (26–39)	41 (30–50)	34 (27–43)	35 (29–41)
Males	36 (31–43)	38 (31–45)	39 (33–47)	39 (33–47)	37 (31–44)	39 (33–47)	36 (29–44)	36 (30–43)

Abbreviations: ART, antiretroviral therapy; IQR, interquartile range; PWH, people with human immunodeficiency virus.

^a^Excluding South Africa.

Across regions, the proportion of PWH with CD4 measured at ART initiation generally increased, or remained stable, from 2005, before declining from around 2013 in the sub-Saharan Africa regions (excluding South Africa) and Latin America (Figure 1*A*). CD4 measurement increased from 58% in 2005 to 73% in 2009 in Southern Africa, from 58% in 2005 to 72% in 2012 in Central Africa, and from 74% in 2005 to 91% in 2012 in West Africa. Over 2005–2012, CD4 measurement remained between 77% and 87% in East Africa and between 87% and 93% in Latin America. Thereafter, CD4 measurement declined sharply in the sub-Saharan African regions and Latin America. From 2009 to 2019 it declined from 73% to 13% in Southern Africa; from 2013 to 2019 it declined from 85% to 31% in East Africa, 72% to 20% in Central Africa, 91% to 53% in West Africa, and 87% to 56% in Latin America. It remained between 68% and 78% from 2005 until 2017 in South Africa, before declining slightly to 61% in 2019. It remained between 80% and 90% in North America throughout the study period. Trends in the Asia-Pacific region were less clear, with fluctuation in proportions of PWH with CD4 measured by year over the whole study period. Our findings changed negligibly (0–4 percentage points) when including CD4 counts measured within 4 or 6 weeks (rather than 2 weeks) after ART initiation (Section S3 of the Supplementary Material).

Among those with a CD4 measured at ART initiation, prevalence of CD4 <200 declined over time in all regions from 2005 (starting at 54% in North America, 90% in South Africa, and 70%–86% in the other regions, and then plateaued after 2015 with the aggregate prevalence of CD4 <200 in 2016–2019 26% in North America; 32%–34% in Southern Africa, East Africa, Central Africa, South Africa, and Latin America; 40% in West Africa; and 47% in Asia-Pacific (Figure 1*B*). Consistent with the trend of decline in the proportions of PWH with CD4 <200, median CD4 count increased across regions from 2005 to 2015 and was 310–348 cells/μL in Southern Africa, East Africa, Central Africa, South Africa, North America, and Latin America; 270 cells/μL in West Africa; and 235 cells/μL in Asia-Pacific in 2019 (Figure 1*C*). All regions showed pronounced increases (of 67% to 2.7-fold) in median and quartile CD4 values from 2005 to 2015, with less uniform and sometimes attenuated trends thereafter.

From 2016 to 2019, the prevalence of CD4 <200, among those with a CD4 count at ART initiation, ranged from 32% to 40% for the 4 Africa regions (excluding South Africa); the prevalence remained high (21%–34%) in exploratory supplementary analyses estimating an approximate lower bound for all PWH initiating ART (Section S2 of the Supplementary Material).

Region-specific CD4 statistics by birth sex-age strata are included in Section S1 of the Supplementary Material. While patterns of CD4 measurement varied among regions, regions consistently showed a higher prevalence of CD4 <200 at older ages through all years, and, in African regions, in males.

## DISCUSSION

Using observational data on 1.35 million PWH aged 15–80 years starting ART at treatment programs from 42 countries that participate in the IeDEA global collaboration, we found that measurement of CD4 count declined rapidly in sub-Saharan African regions, excluding South Africa, and remained fairly constant in other regions. Prevalence of CD4 <200 decreased over time, then plateaued at around 30% or greater in all regions after 2015.

Our findings expand on those reported in previous studies. A regression discontinuity analysis of sites in the global IeDEA collaboration, which considered only data within 2 years of country-level Treat-All adoption, showed that CD4 measurement before first ART initiation decreased from 57% before Treat-All adoption to 48% immediately after in adults in low- and lower-middle-income countries, whereas in high- and upper-middle-income countries it remained high (90% to 92%) [5]. A study from 6 sites in Uganda found that CD4 testing at ART initiation declined from 73% in 2013 to 21% in 2018 [6]. Studies from Botswana, South Africa, Uganda, and China have all reported proportions of new ART initiators with CD4 counts <200 of 20%–30% in the Treat-All era [6, 9–11]. Similar to our findings, some of those studies also found associations between prevalence of CD4 <200 and male sex and older age [9, 10].

We did not aim to identify reasons for the decline in CD4 measurement in our study, but it is possible that decreased funding and/or availability of laboratory equipment or reagents played a role, particularly in lower-resource settings [16–18]. Previous authors have suggested that scale-up of Treat-All policies, with increasing numbers of PWH starting ART, might have placed pressure on limited capacity for testing [19] and that rapid ART initiation for all might have been prioritized over identifying PWH with AHD who needed additional screening and prevention [6]. This might have contributed to the decline in CD4 measurement seen in our study (eg, in Southern Africa), but numbers of new ART initiators did not increase substantially at a regional level in other regions.

Our study's strengths include the large, multiregional, multicountry study population and long period of study (2005–2019), substantially extending results reported in the existing literature. However, using routine clinical data also carries limitations: we might have underestimated CD4 count measurement if tests were performed but not documented. We also excluded data from after 2019, and future studies should explore whether our findings have persisted during and after the end of the COVID-19 pandemic. Our results for the prevalence of CD4 <200 relate to people who presented for HIV care and had CD4 measured at ART initiation, and are not necessarily representative of all PWH initiating ART. However, our simple supplementary analysis using available WHO staging data suggests that within our IeDEA study population, this prevalence is likely to be concerningly high in all PWH initiating ART, which is comparable to other published studies [6, 9–11]. Although outside the scope of this study, future studies should assess the impact of reduced CD4 measurement on treatment outcomes to inform policy and optimal resource allocation.

## CONCLUSIONS

Our findings provide further evidence that measurement of CD4 has declined to very low levels since the Treat-All recommendation, especially in sub-Saharan Africa. Despite initial observed decreases in the prevalence of CD4 <200 at ART initiation at the beginning of the Treat-All era, little change has been observed in the years following, and the ongoing high proportion of PWH with CD4 <200 at ART initiation is of concern. To better monitor and evaluate ART programs, and to guide individual care, CD4 measurement around the time of ART initiation should be more widely adopted and adequately funded as recommended by WHO guidelines.

## Supplementary Material

ciae548_Supplementary_Data

## Supplementary Data

Supplementary materials are available at *Clinical Infectious Diseases* online. Consisting of data provided by the authors to benefit the reader, the posted materials are not copyedited and are the sole responsibility of the authors, so questions or comments should be addressed to the corresponding author.

## Notes

***Author contributions.*** Conceptualization, design, and analysis: R. D. and R. K. Interpretation of results: All authors. Drafting of the original manuscript: R. D. and R. K. Critical review and approval of final manuscript: All authors.

***Data availability.*** Data are not publicly available.

***Disclaimer.*** This work is solely the responsibility of the authors and does not necessarily represent the official views of any of the funding institutions mentioned below.

***Financial support*.** The International epidemiology Databases to Evaluate AIDS (IeDEA) is supported by the US National Institutes of Health's (NIH) National Institute of Allergy and Infectious Diseases, the Eunice Kennedy Shriver National Institute of Child Health and Human Development, the National Cancer Institute, the National Institute of Mental Health, the National Institute on Drug Abuse, the National Heart, Lung, and Blood Institute, the National Institute on Alcohol Abuse and Alcoholism, the National Institute of Diabetes and Digestive and Kidney Diseases, and the Fogarty International Center; grant numbers are as follows: Asia-Pacific, U01AI069907; CCASAnet, U01AI069923; Central Africa, U01AI096299; East Africa, U01AI069911; NA-ACCORD, U01AI069918; Southern Africa, U01AI069924; West Africa, U01AI069919. Informatics resources are supported by the Harmonist project (R24AI24872). V. C. M. has received funding support from the Emory Center for AIDS Research (P30AI050409) for work related to this manuscript.
