# Supplementary material for: Global Trends in CD4 Count Measurement and Distribution at First Antiretroviral Treatment Initiation
Source: Clin Infect Dis. 2024 Nov 6;80(6):1332–6. doi: 10.1093/cid/ciae548 (PMC12264493; doi:10.1093/cid/ciae548)
Supplement: ciae548_Supplementary_Data [file ciae548_supplementary_data.docx]

Supplementary content

This document provides supplementary analysis outputs for the article:

de Waal et al. Global trends in CD4 count measurement and distribution at first antiretroviral treatment initiation. 2024.

**Table of contents**

**Section S1: Characteristics of people with HIV (PWH) starting antiretroviral treatment (ART); and CD4 measurement and distribution overall and by sex-age strata – by year and by region** **2**

S1.1 Southern Africa (excluding South Africa) 3

S1.2 East Africa 4

S1.3 Central Africa 5

S1.4 West Africa 6

S1.5 South Africa 7

S1.6 North America 8

S1.7 Latin America 9

S1.8 Asia-Pacific 10

**Section S2: Approximate lower bound for prevalence of CD4 count < 200 cells/µl in all PWH starting ART during 2016-2019 estimated using WHO staging data, by region 11**

**Section S3: CD4 measurement within varying windows of time around ART start, by region 13**

# Characteristics of people with HIV (PWH) starting antiretroviral treatment (ART); and CD4 measurement and distribution overall and by sex-age strata – by year and by region/country

## Southern Africa (excluding South Africa)

### Characteristics of PWH starting ART, and CD4 measurement and distribution, by year

### CD4 measurement and CD4 < 200 cells/µl, by year of ART start, by age-sex strata

## East Africa

### Characteristics of PWH starting ART, and CD4 measurement and distribution, by year

### CD4 measurement and CD4 < 200 cells/µl, by year of ART start, by age-sex strata

## Central Africa

### Characteristics of PWH starting ART, and CD4 measurement and distribution, by year

### CD4 measurement and CD4 < 200 cells/µl, by year of ART start, by age-sex strata

## West Africa

### Characteristics of PWH starting ART, and CD4 measurement and distribution, by year

### CD4 measurement and CD4 < 200 cells/µl, by year of ART start, by age-sex strata

## South Africa

### Characteristics of PWH starting ART, and CD4 measurement and distribution, by year

### CD4 measurement and CD4 < 200 cells/µl, by year of ART start, by age-sex strata

## North America

### Characteristics of PWH starting ART, and CD4 measurement and distribution, by year

### CD4 measurement and CD4 < 200 cells/µl, by year of ART start, by age-sex strata

## Latin America

### Characteristics of PWH starting ART, and CD4 measurement and distribution, by year

### CD4 measurement and CD4 < 200 cells/µl, by year of ART start, by age-sex strata

## Asia-Pacific

### Characteristics of PWH starting ART, and CD4 measurement and distribution, by year

### CD4 measurement and CD4 < 200 cells/µl, by year of ART start, by age-sex strata

# Approximate lower bound for prevalence of CD4 count < 200 cells/µl in all PWH starting ART during 2016-2019 estimated using WHO staging data, by region

### Prevalence of CD4 < 200 cells/µL

| **Region** | **N with CD4 measured** | **N without CD4 measured** | **Observed prevalence in people with a CD4 result (%)** | **Lower-bound prevalence in all people starting ART, estimated using WHO staging data (%)** |  |
| --- | --- | --- | --- | --- | --- |
| **Southern Africa** | 63820 | 190332 | 32 | 21 |  |
| **East Africa** | 18893 | 30287 | 33 | 24 |  |
| **Central Africa** | 6628 | 7445 | 33 | 31 |  |
| **West Africa** | 3594 | 1421 | 40 | 34 |  |
| All number relate to 2016-2019. Southern Africa excludes the country of South Africa.   - Our study reports the CD4 distribution in PWH who have CD4 measured at ART initiation. However, it is also of public health interest to determine whether the prevalence of CD4 <200 is concerningly high in all PWH initiating ART. We performed a simplistic exploratory supplementary analysis using WHO staging data to produce a lower-bound estimate (i.e. ‘best-case’ scenario estimate) for the prevalence of CD4 <200 in all PWH starting ART. - This simplistic exploratory analysis estimated an approximate lower bound for prevalence of CD4 <200 in all people starting ART during 2016-2019 in our study population (i.e. including those with no documented CD4 result), using available WHO staging data, for the four IeDEA regions with WHO stage at ART start recorded for >60% of people (remaining regions not shown had WHO stage recorded for 0-28% of people). - We assumed that PWH without CD4 measurement had:   ▪ the same prevalence of CD4 < 200 as PWH with CD4 measured who match by region, birth sex (binary), age group (15-24, 25-34, ≥35 years), year of ART start (individual calendar years for the two larger regions, 2016-17 vs 2018-19 for remaining regions) and WHO stage (1, 2, 3 or 4), when WHO stage is recorded;  ▪ CD4 ≥ 200 when WHO stage is not recorded. | | | | | |

### Distribution of WHO stage by whether CD4 was measured

### CD4 measurement and CD4 < 200 cells/µl, by year of ART start, by WHO stage

# CD4 measurement within varying windows of time around ART start, by region

This sensitivity analysis reports the proportion of people who have a documented CD4 count result within 6 months before and 2 (primary analysis), 4 or 6 weeks after documented ART start.

### CD4 measurement within 6 months before and 2/4/6 weeks after ART start

^1^ Excluding South Africa
